# Trends in Telehealth Care During the COVID-19 Pandemic for the Military Health System

**DOI:** 10.1089/tmr.2022.0042

**Published:** 2023-06-26

**Authors:** Thomas Gilder, Amanda Banaag, Cathaleen Madsen, Tracey Pèrez Koehlmoos

**Affiliations:** ^1^ Department of Preventive Medicine and Biostatistics, Uniformed Services University of the Health Sciences, Bethesda, Maryland, USA.; ^2^ The Henry M. Jackson Foundation for the Advancement of Military Medicine, Inc., Bethesda, Maryland, USA.

**Keywords:** electronic health records, medical records, military health, telecommunications, telehealth, telemedicine

## Abstract

**Introduction:**

The COVID-19 pandemic generated a major shift from in-person to telehealth care in efforts to reduce the spread of infection. This study assesses the effects of COVID-19 on the provision of telehealth in the United States Military Health System (MHS), a universally-insured, nationally representative population of beneficiaries who may receive direct care (DC) at military facilities or in the private-sector care (PSC).

**Methods:**

Under a cross-sectional study design, we queried the MHS Data Repository for all telehealth services in the MHS from January 2019 to December 2021, using common procedure terminology code telehealth modifiers GT, GQ, and 95. Analyses were stratified by clinical, provider, and facility characteristics, and comparisons were made between telehealth rates before and during the COVID-19 period using a percent change.

**Results:**

Telehealth usage increased by 20-fold in 2020 versus 2019, whereas provider types shifted from predominantly physicians to advanced practice nurses and physician assistants. Patterns of task shifting were different between DC and PSC. Tele-mental health visits showed a 118% change in DC and −20% change in PSC, suggesting recapture of care to military facilities. Decreases in DC telehealth visits for metabolic, endocrine, and musculoskeletal disorders were not compensated by increases in PSC, suggesting care deferred, delivered by another modality, or sought outside the MHS.

**Conclusion:**

The increase in telehealth usage and behavioral health is in line with other published studies, whereas the shift in provider types aligns with MHS goals focused on increasing access through telehealth. More research is needed to answer questions of care deferral, which are relevant to national health care discussions.

## Introduction

Telehealth, the application of modern communications technology to the practice of medicine, is a feature of many health systems and is valued for its ability to increase access while saving time and costs.^1^ Common modalities of telehealth include synchronous use, such as telephone and virtual visits, asynchronous use such as secure messaging through patient portals, and mobile health applications also known as m-health, some of which can be used for asynchronous monitoring of patients' conditions.^2^

Despite historic barriers to adoption, including access, reimbursement, and regulatory challenges, the percentage of hospitals using telehealth increased from 35% in 2010 to 76% in 2017.^3^ These increases were accelerated by the COVID-19 pandemic, which taxed the American health care system in many previously unanticipated ways. Reliable access to medical advice and some types of evaluation through telehealth meant that patients and health care facilities could minimize risk of exposure, which was particularly important for patients at increased risk.^3^ Practices of specialty care requiring a high degree of interpersonal contact but little physical interaction, such as behavioral health, were able to be completely converted to telehealth visits.^4,5^

Other practices, such as surgical specialties, were able to leverage telehealth for the non-physical aspects of practice such as interprofessional consults and pre- and postoperative encounters.^6,7^ Predictive estimates put telehealth usage as high as 80% of visits in hard-hit areas from March to April 2020,^8^ whereas data from the four largest telehealth providers show visits increasing by 50% during the first quarter of 2020 and up to 154% during week 13 of surveillance, compared with the same periods in 2019, before decreasing and plateauing by November 2020 in response to changing infection rates.^9,10^

Like its non-federal counterparts, the United States Military Health System (MHS) also makes use of telehealth capabilities. This system serves 9.6 million universally-insured beneficiaries, ∼80% non-active duty, who may receive direct care (DC) at military facilities or private-sector care (PSC) through civilian providers who accept the TRICARE insurance benefit. Prior research shows that use of telehealth in the MHS rose 19-fold between 2006 and 2018, driven largely by PSC.^11^ It is expected that telehealth use increased in the MHS during the pandemic as it did in non-federal health systems; however, this has not been conclusively investigated.

This study evaluates the pre-pandemic trends of telehealth use in the MHS beginning in 2019, and changes in usage during the onset and progression of the pandemic. Telehealth use is broken down by provision category (preventive, primary, or subspecialty care), diagnostic category, and beneficiary demographics. Results are expected to inform discussion and provide a context for expansion of telehealth services in the MHS and the nation.

## Methods

### Study population and database

The MHS includes ∼9.6 million universally insured beneficiaries, ∼80% non-active-duty, who may receive DC from military treatment facilities (MTFs) or PSC from civilian providers who accept the TRICARE insurance benefit.^12^ The database is broadly representative of the greater U.S. population between ages 18 and 65, is socioeconomically and demographically diverse, and includes ∼1.9 million children.^13,14^

This does not include care provided by the Veterans Administration, which is a separately administered system, or care provided in forward deployed environments, although it does include MTF-based care provided outside the continental United States. Claims data for MHS beneficiaries using both DC and PSC are captured in the MHS Data Repository (MDR), a series of linked databases that has been used in over 100 published studies. This study was decreed exempt from human subject oversight by the Institutional Review Board of the Uniformed Services University of the Health Sciences.

### Data acquisition and management

Under a cross-sectional study design, we queried the MDR for all telehealth services in the MHS from January 2019 to December 2021. Eligibility for the study population includes all TRICARE Prime/Prime Plus beneficiaries (∼5 million active duty, dependent, and civilian beneficiaries),^11^ with at least one telehealth encounter in DC or PSC capacities. Telehealth encounters were identified by using common procedure terminology codes GT for synchronous telehealth, GQ for asynchronous telehealth, or 95 for services taking place via telehealth.^11^

Telehealth visits were defined as having at least one telehealth service per patient per day; if a patient had multiple telehealth services per day, we counted it as one visit. Provider type and specialty, facility care setting and geographic location, and delivered care characteristics (synchronous vs. asynchronous service, and major diagnostic category) were obtained from the telehealth visit and used in analyses.

### Analysis

Descriptive statistics were used to assess trends in telehealth encounter rates—defined as per 1000 encounters during the referenced time period—and percent change was used to assess changes in rates. Analyses were stratified by clinical, provider, and facility characteristics, and comparisons were made between telehealth rates before and during the COVID-19 period. Descriptive statistics of telehealth care events by provider specialty and skill were performed to evaluate the uptake in telehealth appointments by provider skill type. Primary data cut and analysis was performed using SAS 9.4 (Cary, NC).

## Results

Overall, the number of telehealth visits recorded in the MHS increased during the COVID-19 period, in both the DC and PSC arenas (Table 1). DC visits rose from 111,368 in 2019 to a peak of 294,098 in 2020, before dropping to 193,526 visits in 2021. In contrast, PSC visits rose from 26,770 in 2019 to a high of 2,614,409 visits in 2022. The greatest number of total telehealth visits was 2,891,865 in 2020, a 20-fold increase over the previous year.

**Table 1.** Telehealth Visits in the Military Health System, 2019–2021

*Calendar year*	*DC*				*PSC*			
	*Telehealth claims*	*Total number of claims*	*Prevalence rate*	*% Change in number of claims*	*Telehealth claims*	*Total number of claims*	*Prevalence rate*	*% Change in number of claims*
2019	111,368	29,610,636	3.76	298.65	26,770	26,451,716	1.01	29.14
2020	294,089	25,430,098	11.56	164.07	2,597,776	26,472,071	98.13	9604.06
2021	193,526	23,910,700	8.09	−34.19	2,614,409	29,376,190	89.00	0.64

DC, direct care; PSC, private-sector care.

This also represented an increased prevalence rate of telehealth visits among total health care visits in both care arenas; although total health care visits also peaked in 2020, telehealth visits represented a greater proportion of those encounters, rising from prevalence rates of 3.76 in 2019 to 11.56 in 2020 in the DC setting and from 1.01 in 2019 to 98.13 in PSC. This study also compared changes in synchronous and asynchronous telehealth during the COVID period to the pre-COVID period, for DC and PSC.

Synchronous telehealth increased with a 2.60% change in DC and a 1.92% change in PSC during the COVID period, whereas asynchronous telehealth decreased with a −19.23% change in DC and −72.48% change in PSC during the COVID period (Table 2).

**Table 2.** Synchronous Versus Asynchronous Telehealth Services

*Telehealth delivery type*	*DC*					*PSC*				
	*Pre-COVID19 (***n*** = 111,368)*		*During COVID-19 (***n*** = 487,615)*		*% Change*	*Pre-COVID 19 (***n*** = 26,770)*		*During COVID-19 (***n*** = 5,212,185)*		*% Change*
	*Count*	*% of Column ***N****	*Count*	*% of Column ***N****		*Count*	*% of Column ***N****	*Count*	*% of Column ***N****	
Asynchronous	13,267	11.91	46,895	9.62	−19.23	691	2.58	37,013	0.71	−72.48
Synchronous	98,101	88.09	440,720	90.38	2.60	26,079	97.42	5,175,172	99.29	1.92

### Provider types

In DC during the pre-COVID period, the largest proportion of telehealth delivery was performed by Doctor of Medicine (MD) or Doctor of Osteopathy (DO) clinicians (54.27%), followed by advanced practice nurses (APNs) and physician assistants (PAs; 44.80%), as shown in Table 3. During the COVID-19 period, the pattern was reversed, with APNs/PAs performing 55.72%, and MD/DOs performing 39.40%. of telehealth visits.

**Table 3.** Relative Trends and Percent Change in Telehealth by Provider Type

*Provider skill level*	*DC*					*PSC*				
	*Pre-COVID19 (***n*** = 111,368)*		*During COVID-19 (***n*** = 487,615)*		*% Change*	*Pre-COVID 19 (***n*** = 26,770)*		*During COVID-19 (***n*** = 5,212,185)*		*% Change*
	*Count*	*% of Column ***N****	*Count*	*% of Column ***N****		*Count*	*% of Column ***N****	*Count*	*% of Column ***N****	
APN/PA	49,898	44.80	271,686	55.72	24.38	12,562	46.93	2,645,976	50.77	8.18
Clinician (MD/DO)	60,437	54.27	192,126	39.40	−27.40	9149	34.18	1,621,890	31.12	−8.95
Para-professional	385	0.35	19,819	4.06	1060.00	4585	17.13	766,004	14.70	−14.19
Registered nurses	636	0.57	3513	0.72	26.32	6	0.02	698	0.01	−50.00
Administrative	12	0.01	471	0.10	900.00	223	0.83	61,976	1.19	43.37
Facility	0	0.00	0	0.00	—	245	0.92	115,641	2.22	141.30
Other (para-professional and admin)	397	3.49	20,290	4.16	5010.83	4808	17.96	827,980	15.89	17,120.88

APN, advanced practice nurse; DO, Doctor of Osteopathy; MD, Doctor of Medicine; PA, physician assistant.

In PSC, the APNs/PAs performed the majority of telehealth care during both pre-COVID and during-COVID periods, but it increased their relative proportions of care provided. (46.93% increased to 50.77% of telehealth visits, respectively). MD/DOs in DC performed 54.27% of telehealth visits in the pre-COVID period, decreasing to 39.40% during COVID; whereas in PSC, they performed 34.18% of pre-COVID visits, decreasing to 31.12% of visits during COVID.

Paraprofessionals, which include psychologists, licensed clinical social workers, occupational and physical therapists, and other similarly trained professionals who do not hold nursing or physician degrees, and administrators in DC showed large % changes (1060 and 900, respectively) in visits provided before and during COVID, likely due to small sample sizes.

**FIG. 1.**
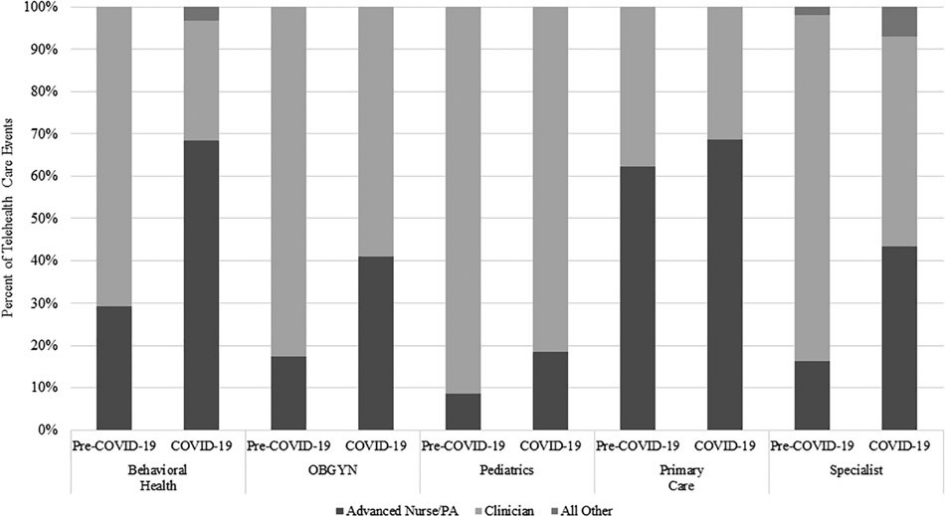
Shift in telehealth care events during the COVID-19 pandemic in the MHS by provider specialty and skill type, direct care. MHS, Military Health System; OBGYN, Obstetrics and Gynecology; PA, physician assistant.

**FIG. 2.**
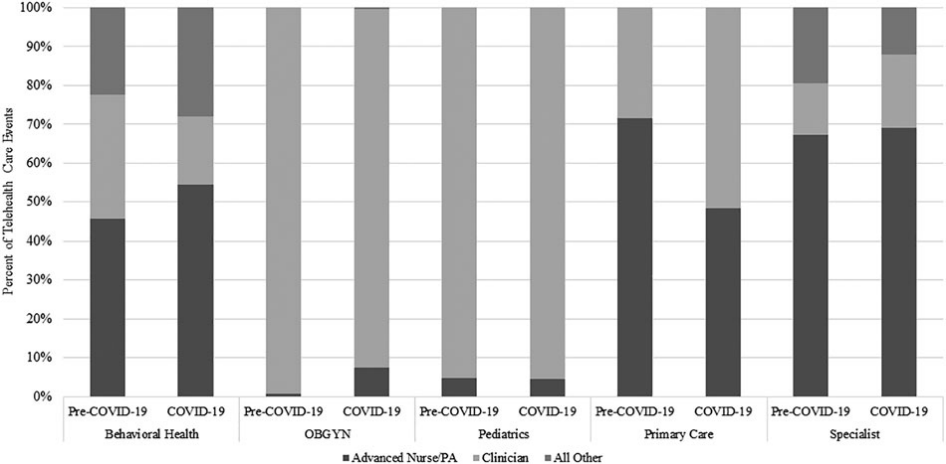
Shift in telehealth care events during the COVID-19 pandemic in the MHS by provider specialty and skill type, private sector care.

To determine deeper patterns in the task shifting of providers from MD/DO to APN/PA, the above results were further stratified by medical specialty, in both DC (Fig. 1) and PSC (Fig. 2). The results in DC showed that this shift was consistent across the board, whereas results in PSC differed in two categories. Pediatrics showed a small (<5%) decrease and Primary Care ∼a 20% decrease in the percentage of telehealth visits provided by APN/PA compared with pre-pandemic numbers (Fig. 2).

Notably, the percentage of behavioral health visits provided by clinicians decreased in both DC and PSC, with additional visits shifted to “All Other” providers who were neither clinicians nor APN/PA. This pattern was also seen in specialist care in DC, but not PSC.

### Medical specialty and diagnoses

The type of specialty care and diagnoses also changed during COVID in both DC and PSC. In DC, the majority of telehealth visits were for Primary Care in both the pre-COVID and during-COVID periods (63.19% and 50.26%, respectively); however, their proportion of the total telehealth visits decreased during COVID (−20.46% change). In contrast, Behavioral Health in DC increased from 4.99% pre-COVID to 21.81% for a % change of 337.07.

In PSC, the majority of telehealth visits were for Behavioral Health in both pre-COVID (76.50%) and during-COVID periods (54.89%), though again the proportion decreased during COVID (−28.25). In contrast, Specialist Care in COVID increased from 8.39% pre-COVID to 20.41%, for a % change of 143.27. Preventive Medicine notably declined in DC (−43.56% change) and increased in PSC (135.90% change) during COVID, suggesting some outsourcing of care to the private sector. Large positive or negative % changes in other specialist categories are reflective of effects on small sample sizes (Table 4).

**Table 4.** Relative Trends and Percent Change in Telehealth by Specialty

*Provider specialty*	*DC*					*PSC*				
	*Pre-COVID19 (***n*** = 111,368)*		*During COVID-19 (***n*** = 487,615)*		*% Change*	*Pre-COVID 19 (***n*** = 26,770)*		*During COVID-19 (***n*** = 5,212,185)*		*% Change*
	*Count*	*% of Column ***N****	*Count*	*% of Column ***N****		*Count*	*% of Column ***N****	*Count*	*% of Column ***N****	
Primary care	70,375	63.19	24,5052	50.26	−20.46	2353	8.79	715,434	13.73	56.20
Preventive medicine	15,747	14.14	38,936	7.98	−43.56	834	3.12	383,623	7.36	135.90
Pediatrics	10,098	9.07	32,352	6.63	−26.90	661	2.47	140,006	2.69	8.91
Specialist	7300	6.55	40,862	8.38	27.94	2245	8.39	1,063,907	20.41	143.27
Behavioral health	5552	4.99	106,366	21.81	337.07	20,480	76.50	2,861,036	54.89	−28.25
Other	1170	1.05	20,827	4.27	306.67	38	0.14	15,128	0.29	107.14
OBGYN	1126	1.01	3220	0.66	−34.65	159	0.59	33,051	0.63	6.78

OBGYN, obstetrics and gynecology.

In DC, the most common pre-COVID diagnosis was Factors Influencing Health Status and Other Contacts with Health Services, which category includes service members' regularly scheduled health screenings, and accounted for 21.97% of telehealth encounters (Table 5). This was also the second most common diagnosis in DC during COVID at 20.20% of telehealth encounters. The most common during-COVID diagnosis was Mental Diseases and Disorders at 21.47% of telehealth encounters, a 118.64% change from pre-COVID values. This was also the most common diagnosis in PSC both before and during COVID; however, the values decreased during COVID (−20.00% change).

**Table 5.** Relative Trends and Percent Change in Telehealth by Major Diagnostic Category

*Major diagnostic category*	*DC*					*PSC*				
	*Pre-COVID19 (***n*** = 111,368)*		*During COVID-19 (***n*** = 487,615)*		*% Change*	*Pre-COVID 19 (***n*** = 26,770)*		*During COVID-19 (***n*** = 5,212,185)*		*% Change*
	*Count*	*% of Column ***N****	*Count*	*% of Column ***N****		*Count*	*% of Column ***N****	*Count*	*% of Column ***N****	
Factors influencing health status and other contacts with health services	24,470	21.97	98,515	20.20	−8.06	374	1.40	109,754	2.11	50.71
DoD unique codes (used only in CAPER) which are unique attributes, not ICD codes	13,162	11.82	21,563	4.42	−62.61	—	—	—	—	
Endocrine, nutritional, and metabolic diseases and disorders	13,058	11.73	28,642	5.87	−49.96	301	1.12	244,676	4.69	318.75
Diseases and disorders of the musculoskeletal system and connective tissue	12,561	11.28	46,881	9.61	−14.80	187	0.70	252,570	4.85	592.25
Mental diseases and disorders	10,940	9.82	104,691	21.47	118.64	22,414	83.73	3,491,271	66.98	−20.00
Diseases and disorders of the circulatory system	5131	4.61	18,697	3.83	−16.92	118	0.44	87,341	1.68	281.82
Diseases and disorders of the skin, subcutaneous tissue, and breast	4370	3.92	27,553	5.65	44.13	261	0.97	90,666	1.74	79.38
Diseases and disorders of the ear, nose, mouth, and throat	4281	3.84	24,271	4.98	29.69	794	2.97	198,672	3.81	28.28
Diseases and disorders of the digestive system	3912	3.51	17,431	3.57	1.71	160	0.60	133,069	2.55	325.51
Diseases and disorders of the nervous system	3627	3.26	22,415	4.60	41.10	524	1.96	219,914	4.22	115.27
Diseases and disorders of the female reproductive system	3414	3.07	13,499	2.77	−9.77	143	0.53	45,901	0.88	66.04
Diseases and disorders of the kidney and urinary tract	3234	2.90	9660	1.98	−31.72	88	0.33	47,078	0.90	172.73
Diseases and disorders of the respiratory system	2955	2.65	13,785	2.83	6.79	211	0.79	113,393	2.18	175.38
Diseases and disorders of the blood, blood-forming organs, immunological disorders	1768	1.59	3457	0.71	−55.35	34	0.13	20,547	0.39	200.00
Infectious and parasitic diseases, systemic or unspecified sites	898	0.81	6786	1.39	71.60	65	0.24	56,066	1.08	350.00
Pregnancy, childbirth, and the puerperium	886	0.80	2781	0.57	−28.75	69	0.26	13,737	0.26	0.00
Diseases and disorders of the male reproductive system	775	0.70	1851	0.38	−45.71	19	0.07	11,111	0.21	200.00
Diseases and disorders of the hepatobiliary system and pancreas	740	0.66	1468	0.30	−54.55	11	0.04	10,421	0.20	400.00
Diseases and disorders of the eye	583	0.52	3506	0.72	38.46	468	1.75	14,958	0.29	−83.43
Injuries, poisonings, and toxic effects of drugs	201	0.18	1008	0.21	16.67	34	0.13	5693	0.11	−15.38
Alcohol/drug use and alcohol/drug-induced organic mental disorders	167	0.15	16,891	3.46	2206.67	470	1.76	34,055	0.65	−63.07
Newborns and other neonates with conditions originating in perinatal period	101	0.09	391	0.08	−11.11	13	0.05	4746	0.09	80.00

CAPER, Comprehensive Ambulatory/Professional Encounter Record; DoD, Department of Defense; ICD, International Classification of Diseases.

The second most common diagnosis in PSC during COVID was Diseases and Disorders of the Musculoskeletal System and Connective Tissue with 4.85% of encounters and a 592% change from pre-COVID values, indicating that overall increase in telehealth usage is reflected in small changes across a variety of categories.

## Discussion

This study identified significant transitions in the patterns of telehealth usage pre- and during-COVID in the MHS. Given the focus on maintaining social distance to reduce infection, particularly in the early days of the pandemic, it is not surprising that the number of telehealth encounters increased ∼20-fold in 2020. This reflects the trends observed in other American health care systems, with Medicare reporting a 430-fold increase in telehealth for primary care (from 0.1% to 43%) during February–April 2020^15^ and private insurers reporting a 79-fold increase (0.3% to 23.6%) in all care contacts from 2019 to March–June 2020.^16^

These studies pool synchronous and asynchronous contacts rather than separating them as in our study, making direct comparison difficult. Similarly, while both articles describe the utility of APN/PA and physician extenders to provide telehealth care, the Medicare study focuses on changes to privileging and reimbursement, while the study in private insurance reports changes in terms of provider load versus percentage of providers delivering telehealth as in our study.

The overall shift in provider types delivering telehealth during the COVID-19 pandemic appears understudied in literature. However, the results of this study demonstrate an alignment with previously-published MHS goals of using non-physician providers on a telehealth platform to extend clinical reach, potentially reducing costs while preserving quality.^11^

This is important not just for the MHS, but also for the civilian health care systems to which MHS providers delivered support during the pandemic, despite the MHS staffing shortages.^17^ Greater need for intensive, hands-on, physician care in either system is a likely contributor to decreased physician availability to provide telehealth services. However, in-person care was not assessed for this study, and care provided outside the MHS, even if by MHS providers, is not captured in our data set, which factors limit full discussion of these findings in the context of clinical acuity.

One notable factor is the increase in percentage of telehealth visits provided by “All Other” category health care providers in behavioral health (DC and PSC) and in specialist care (DC). As described in Table 3, the “All Other” category refers to paraprofessionals, registered nurses other than APNs, and any administrators or facility staff associated with the patient's care.

In behavioral health, this category could also include counselors, licensed clinical social workers, and doctorally prepared psychologists who do not hold an MD or DO. In specialist care, this could include the many technicians who practice under physician oversight, such as respiratory therapists, registered dieticians, or physical and occupational therapists, particularly for routine monitoring that does not require in-person care. Further research is necessary to investigate this pattern more deeply.

Another notable pattern occurs with the increased percentage of telehealth provided by physicians in pediatrics and primary care, which was seen in PSC but not in DC. The percentage of primary care visits provided by telehealth increased overall in PSC but not in DC, which is a likely factor in the greater percentage of care provided by physicians. The percentage of pediatric visits provided by telehealth stayed roughly the same, with a much smaller increase (<5%) in care provided by physicians.

Other categories of care that also showed a large, positive percent change in PSC were not accompanied by an increase in physician-provided telehealth, but as described in Results, these large percent changes appear to be driven by small sample size. As specific delineation of roles and clinical acuity were not captured in this study, further research is necessary to investigate the factors that drive task-shifting in telehealth care during the pandemic.

As noted in Results, the diagnoses associated with telehealth also changed substantially during the study period. Mental Diseases and Disorders in DC increased from 9.82% before COVID-19 to 21.47% during COVID-19, a 119% change. This diagnosis accounted for the largest percent of telehealth usage in PSC both before and during COVID-19, though that usage decreased from 83.73% to 66.98%, for a −20% change.

Because the decrease in PSC was concurrent with an increase in DC for this diagnosis, the change may represent recapture of care by the DC system, either due to increased telehealth resources in DC or to increase in non-MHS patients seeking care in the private sector. In contrast, telehealth usage for Endocrine, Nutrition, and Metabolic Disease, and Diseases and Disorders of the Musculoskeletal System and Connective Tissue decreased in DC during COVID-19 (−49.96% change and 14.80% change, respectively) but their increased usage in PSC appears to be driven by small sample sizes (*n* < 500).

Therefore, the decrease in DC likely represents care deferred,^18,19^ delivered in person, or sought outside the MHS. Although deferral of care is normally discussed in terms of in-person visits, it is possible that telehealth visits were deferred by providers due to workload, or by patients due to increased family stress, reduced access to bandwidth,^20,21^ or lack of privacy, particularly in the early days of the pandemic.

This poses a significant concern for those with chronic conditions such as diabetes or arthritis that must be carefully managed to avoid further disability. The shift from in-person care to telehealth care in both sectors may have been driven by either providers or patients, and this is not captured in our data set. Further research is needed to answer this question.

Mental health has been a particular concern during COVID-19, with adverse mental health reported worldwide due to direct neurologic effects of the virus, public health measures such as social distancing, quarantines, and lockdowns,^22^ and the resulting financial insecurity and lack of access to resources.^13,22,23^ An increase in mental health diagnoses in the MHS would be strongly in line with other published reports; however, some encounters may have taken place outside of a telehealth modality (i.e., in person) or outside the MHS, which would not be captured in this study. Further research is necessary to determine the full effects of COVID-19 on mental health diagnosis and treatment in the MHS.

Another important factor driving patterns of care in the MHS is the interplay between MHS priorities, provider workload, and market forces. While MHS beneficiaries may receive either DC or PSC, in practice, active-duty service members are increasingly prioritized to receive DC,^24^ and beneficiaries may have little choice in the arena that provides their care. Physician workload may also be a factor in both sectors.

The provider shortage has been well documented in the public press as well as scholarly literature, and in the MHS particularly, a decreasing number of physicians both cared for the growing MHS population and provided support to the civilian health care sector during the pandemic.^17^ Taken together, these factors are likely to push care into the private sector, where the providers use a fee-for-service care model instead of the salaried model used in DC. A full investigation of this interplay and its effects on telehealth care are beyond the scope of this paper, and present significant opportunity for further research.

### Strengths and limitations

This study has several notable strengths. The first is the size of the study, at over 5 million visits. The second is the data from a socioeconomically and demographically diverse, nationally representative, substantially working-age population, in contrast to Medicare studies that preferentially include the elderly, or private insurance studies that preferentially include middle and upper-middle income beneficiaries.

This suggests that findings of this study are broadly generalizable to the greater U.S. population. The use of claims data represents both a strength and a limitation. As a strength, it is not subject to recall bias and other limitations as with survey data. As a limitation, it is subject to potential coding errors that are known to occur in large databases. Another limitation is that specific procedures and outcomes were not assessed in this study.

As previously described, clear delineation of roles between physicians, APN/PA, and other providers were not assessed in this study, nor were clinical notes available to assess nuances of case acuity that potentially could route patients between provider tiers or care models. Finally, race was not captured in this study, which limits applicability of results to specific racial and ethnic groups.

## Conclusions

Significant changes in telehealth usage in the MHS during COVID-19 occurred in both DC and PSC, with a 20-fold increase seen in 2020. This study showed a shift from telehealth provided by MD and DO to APN/PA providers, in line with MHS goals to expand provider reach and access through telehealth. A decrease in mental health services in PSC with concurrent increase in DC suggests recapture of care, whereas decreases in DC for endocrine, metabolic, and musculoskeletal disorders were not matched by increased telehealth care in PSC, suggesting deferral of care, care provided by other modalities, or care outside the MHS. Further research is needed to determine these factors.

## Abbreviations Used

APNs: advanced practice nurses
CAPER: Comprehensive Ambulatory/Professional Encounter Record
DC: direct care
DO: Doctor of Osteopathy
DoD: Department of Defense
ICD: International Classification of Diseases
MD: Doctor of Medicine
MDR: MHS Data Repository
MHS: Military Health System
MTFs: military treatment facilities
OBGYN: obstetrics and gynecology
PA: physician assistant
PSC: private-sector care
